# Characterization of the Microstructure and Interfacial Morphology of Magnetic Pulse Welded Steel/Al Tubes

**DOI:** 10.3390/ma18040757

**Published:** 2025-02-08

**Authors:** Tianhan Hu, Bolong Li, Tianhai Wu, Hua Pan, Kai Ding, Yulai Gao

**Affiliations:** 1 State Key Laboratory of Advanced Special Steel, School of Materials Science and Engineering, Shanghai University, Shanghai 200444, China; hutianhan@shu.edu.cn (T.H.); libolong_111@shu.edu.cn (B.L.); dingkaiwsj@shu.edu.cn (K.D.); 2 State Key Laboratory of Development and Application Technology of Automotive Steels (Baosteel Group), Shanghai 201900, China; wuth@baosteel.com; 3 Automobile Steel Research Institute, R&D Center, Baoshan Iron & Steel Co., Ltd., Shanghai 201900, China

**Keywords:** magnetic pulse welding, steel/Al tube, wavy interface, transition zone, collision velocity and angle

## Abstract

Facing the global energy crisis and increasingly stringent environmental protection regulations, automotive lightweighting has become a core issue for the sustainable development of the automotive industry. In particular, the qualified combination of steel and aluminum alloy has become a promising development direction to achieve the aim of lightweight design. As an innovative solid-phase welding technique, magnetic pulse welding (MPW) exhibits unique advantages in joining these dissimilar metals. The 6061 Al alloy and 20# steel tubes were joined by the MPW technique in this study. The microstructure and interface morphology of the MPW steel/Al tube were characterized using optical microscopy (OM), scanning electron microscopy (SEM), and an electro-probe microanalyzer (EPMA). The microstructure in the region adjacent to the interface was similar to that of the base metals (BMs). The element transition zone could be observed at the interface. The thickness of the transition layer was approximately 6 μm. The transition layer did not possess high hardness and brittleness like the Fe–Al binary IMC layer. Therefore, the interface bonding quality and long-term stability of the MPW steel/Al joint were relatively good. The welded joint interface could be divided into three zones: the bonded zone in the center and unbonded zones on both sides. In particular, an obvious wavy interface with gradually increased amplitude was detected in the bonded zone. The interaction between the reflected wave and the welding collision point could promote the initiation of the wavy interface. In addition, the formation of the wavy interface depended on the impact velocity and angle of the MPW process. The qualified mechanical properties of the joint could be attributed to the formation of the wavy interface. The microhardness at the interface was higher than that on both sides, owing to work hardening, at approximately 226 HV.

## 1. Introduction

Global car ownership has increased steadily in recent years. Although they bring convenience to people, automobiles have become one of the main sources of resource consumption and pollutant emissions [1,2,3]. Thus, lightweighting, as an effective way to reduce energy consumption and pollution, has become a hot spot in automobile industry upgrading [4]. Steel is the primary material used for automobile bodies. Currently, advanced high-strength steel (AHSS) is extensively utilized in the load-bearing components of a vehicle body to ensure essential collision safety and structural strength. The first generation of AHSS mainly consists of dual-phase steel (DP), transformation-induced plasticity steel (TRIP), etc. The second generation of AHSS is typified by twinning-induced plasticity steel (TWIP). The third generation of AHSS mainly includes quenching and partitioning steel (QP) and medium Mn steel [5]. The aluminum alloys, especially the 5 and 6 series (according to the ASTM), are widely used to replace steel in some parts of the car body based on their low density and good formability [6]. Thus, the steel/Al dissimilar joints are anticipated to be widely applied in the field of automobile body manufacturing due to their advantages in weight reduction and comprehensive properties.

However, owing to the differences in physical and chemical properties, such as melting point, thermal conductivity, and elastic modulus, achieving steel/Al dissimilar joints with relatively high bonding strength is difficult [7,8]. In particular, it is difficult to avoid the formation of brittle Fe–Al intermetallic compounds (IMCs) during welding, which can strongly affect the mechanical properties of the welded joint [9,10,11]. Several studies [12,13] showed that the excessive growth of the IMC layer could easily induce cracks at the interface. Thus, effectively controlling the formation and growth of the IMC layer becomes a crucial point in steel/Al dissimilar metal welding. At present, the traditional techniques used for steel/Al welding are resistance spot welding (RSW) [14,15], cold metal transfer (CMT) [16,17], melt inert-gas welding (MIG) [18,19], and laser welding [20,21,22]. Unfortunately, this kind of fusion welding cannot easily avoid the formation of IMC, although the welding process has been optimized [23]. As a result, novel welding techniques are urgently needed for steel/Al dissimilar metals.

As an innovative solid-phase welding technique, magnetic pulse welding (MPW), based on the magnetic pulse forming principle, exhibits unique advantages in joining dissimilar metals [24,25]. It is mainly used for welding metal plates and metal tubes. With the high-speed collision of the base metals (BMs) in the magnetic field, metallurgical bonding at the interface can be achieved without melting the jointed parts [26,27]. Thus, the growth of the IMC layer could be effectively limited or even prevented because of the relatively low heat input [28]. In addition, the characteristics of the heat-affected zone (HAZ) are rarely detected in such steel/Al MPW joints [23]. To further improve the properties of the steel/Al MPW joint, the welding process was studied. Kore et al. [29] fabricated a steel/Al MPW joint by adjusting the geometry of the coil, and found that reducing the coil cross-sectional area increased the current density, thereby enhancing the tensile strength of the joint. Yu et al. [30] investigated the influence of coil turns on the strength of welded joints and energy utilization efficiency. The results showed that when a seven-turn coil was used to join t 1060 aluminum alloy and DP600 steel, both the joint strength and energy utilization efficiency were higher. The welding process was more stable. Yao et al. [31] pointed out that the large working area required by traditional coils was not conducive to the further development of the magnetic pulse welding technique. Thus, a miniaturized S-shaped three-dimensional coil was correspondingly designed, which could reduce the working area required by the coil and improve the flexibility of the magnetic pulse welding technique. Resultantly, qualified steel/aluminum welded joints were successfully prepared using this new coil structure. Psyk et al. [32] noted that the quality of the MPW joint could be affected by the base metal spacing, lap length, and capacitance energy. Among these, the spacing of the BMs affected both the collision velocity and collision angle. In addition, the field shaper is one of the most important components of an MPW system. Yan et al. [33] applied a multi-seam field shaper to regulate the distribution of the induced current and the magnetic field, effectively solving the non-uniform deformation of the tubes during welding.

The related studies on steel/Al MPW joints focused on the interface, which is critical to joint properties. In general, the microstructure around the steel/Al MPW joint interface was found to be similar to that of the BMs. However, the transition zone and IMC layer could sometimes be detected there. Geng et al. [34] revealed an amorphous structure and transition zone at the interface of steel/Al dissimilar material joint. Zhang et al. [35] systematically studied the morphology characteristics of the interface of the MPW joints for 6061 aluminum alloy and 304 stainless steel. The IMC layer was detected at the steel/Al interface. Energy-dispersive spectrometer (EDS) and X-ray diffraction (XRD) were used to analyze the phases of the IMC. The results indicate that the IMC consisted of Fe_2_Al_5_ and Fe_4_Al_13_ phases. Stern et al. [36] pointed out that the Fe content in the Fe–Al IMC depended on the induced energy. The interface morphology was particularly important for joint properties, and the wavy interface tended to produce better bonding effects than the flat one [37]. In addition, the impact velocity and angle were the key factors affecting the interface morphology of the joints [38]. Cui et al. [39] found that an increase in discharge energy could accelerate the impact of a flying plate, promoting the transition of the interface from a sinusoidal profile to a shear wave. This transition could improve the reliability of the steel/Al MPW joint. Hahn et al. [40] performed a peel test on the aluminum alloy MPW joint, and the results show that the wavy interface enhanced the joint strength.

In the present study, 6061 aluminum alloy–20# steel dissimilar welded joints were prepared by the magnetic pulse welding process. The microstructure and interface morphology were characterized. The element distribution in the interface between the 6061 aluminum alloy (according to the ASTM) and 20# steel (GB/T 699-2015; Quality carbon structure steel. Beijing, China, 2016.) was analyzed to study the diffusion of elements Fe and Al. In addition, the formation mechanism of the wavy interface was systematically analyzed and is here discussed.

## 2. Experimental Procedures

**Figure 1:**
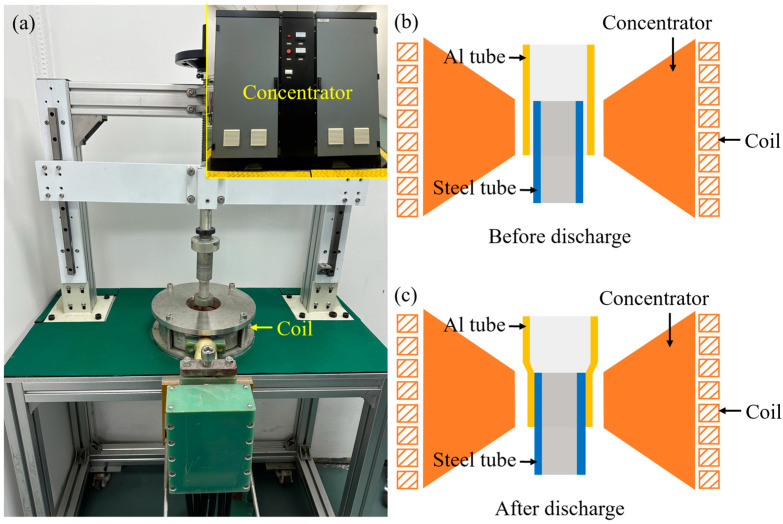
The appearance of the MPW equipment and schematic of the principle for the MPW technique: (**a**) the appearance of the MPW technique; (**b**) MPW steel/Al tubes before discharge; (**c**) MPW steel/Al tubes after discharge.

The outer tube was made of 6061 aluminum alloy. Its outer diameter was 60 mm and the inside diameter was 56 mm. The inner tube was made of 20# steel. The outer diameter was 53 mm, and the inside diameter was 44 mm. The chemical composition of 20# steel and 6061 aluminum alloy is listed in Table 1. The steel/Al tubes were prepared by the magnetic pulse welding (MPW) technique. The electrical energy stored in the welding equipment was converted into the kinetic energy of the BMs. The Al alloy and steel collided at high speed, and thus the Fe and Al elements were able to diffuse rapidly to form the transition zone. Eventually, the steel/aluminum dissimilar metals were successfully joined together. During the welding process, the input voltage was 13.5 kV, the discharge energy was 28 kJ, the maximum voltage was 18 kV, the capacitance was 30 μF, and the total energy was 50 kJ. The appearance of the MPW equipment and a schematic of the principle for the MPW technique are shown in Figure 1.

The sample containing the whole interface between the steel and Al tubes was obtained by the wire-electrode discharge machining (WEDM) method. The specimens were etched after being ground and polished. Considering the different corrosion resistances between 6061 aluminum alloy and 20# steel, a two-step etching method was applied to reveal the dissimilar metal joint. The etchant of HNO_3_ (Taicang Hushi Reagent Co., Ltd., Suzhou, China) + C_2_H_5_OH (Shanghai Wokai Biological Technology Co., Ltd., Shanghai, China) with a volume proportion of 5:1 was applied to reveal the microstructure of 20# steel. Keller’s reagent (HF + HCl + HNO_3_ + H_2_O with the volume proportion of 1:1:1:6, Phygene, Fuzhou, China) was applied to exhibit the microstructure of 6061 aluminum alloy. The microstructure and interface morphology of the steel/Al tube MPW joint were observed by optical microscopy (OM, Zeiss Imager A2m, Zeiss, Oberkochen, Germany) and scanning electron microscopy (SEM, Phenom ProX, Phenom-World, Eindhoven, The Netherland). The elemental distribution was obtained by use of an electro-probe microanalyzer (EPMA, EPMA-8050G, Shimadzu Corporation, Kyoto, Japan). A microhardness test of the joint was performed on a digital microhardness tester (MH-5L, Hengyi Precision Instrument Co., Ltd., Shanghai, China) with a dwell time of 5 s. The applied loads for steel and aluminum alloy were 200 gf and 50 gf, respectively.

## 3. Results

**Figure 2:**
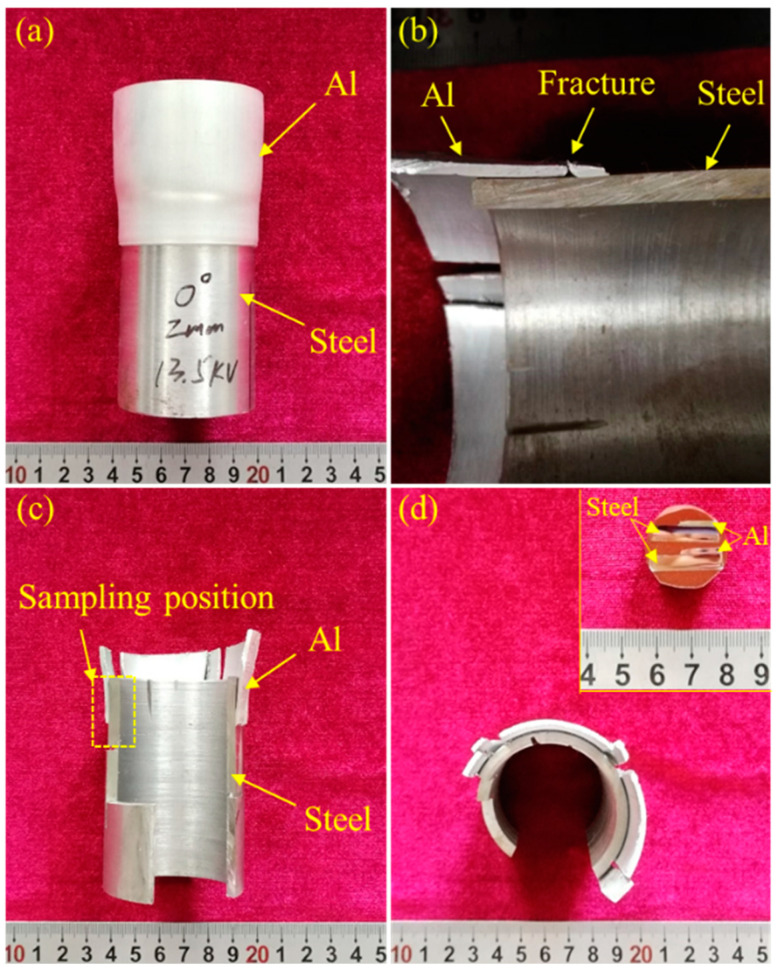
The appearance of the magnetic pulse welded steel/Al tube: (**a**) before peel test; (**b**–**d**) after peel test.

The appearance of the magnetic pulse welded steel/Al tube before and after the peel test is presented in Figure 2. Clearly, the outer Al tube and the inner steel tube were effectively bonded together by magnetic pulse welding (see Figure 2a). No interface failure occurred in the steel/Al MPW joint during the peel test process, reflecting the qualified welding of the MPW joint with the employed welding parameters (see Figure 2b). To shed light on the microhardness, the microstructure and interface morphology of the 6061 aluminum alloy–20# steel MPW joint were further investigated.

**Figure 3:**
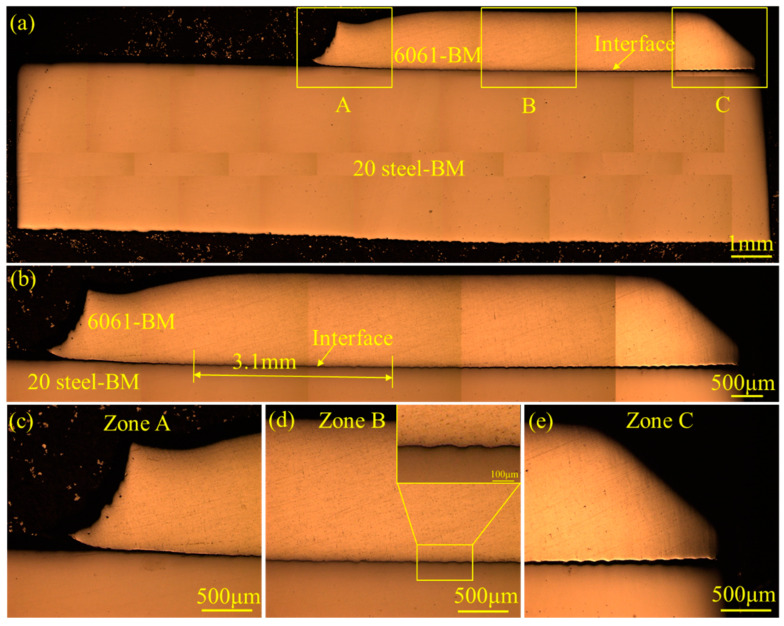
Metallographical images of the 6061 aluminum alloy–20# steel dissimilar welded joint without chemical etching: (**a**) whole macrostructure of the joint; (**b**) whole macrostructure of the interface; (**c**–**e**) magnified images of zones A, B and C, respectively. No interface bonding could be observed in the initial contact part of the outer and inner tubes (see (**c**)) or in the last contact part (see (**e**)). A wavy interface morphology can be seen in the intermediate part of the interface, as shown in (**d**).

The microstructure of the whole joint is shown in Figure 3a. No obvious deformation was found in either the Al or steel tubes. Figure 3b shows the microstructure of the steel–Al interface. The bonding length of the 6061-BM and 20# steel–BM was around 3.1 mm. The metallographical images of the interface at the intermediate and side parts are displayed in Figure 3c,d respectively. No interfacial bonding could be observed in the initial contact part of the outer and inner tubes (see Figure 3c) or in the last contact part (see Figure 3e). In particular, the wavy interface morphology could be found in the intermediate part of the interface, as shown in Figure 3d.

**Figure 4:**
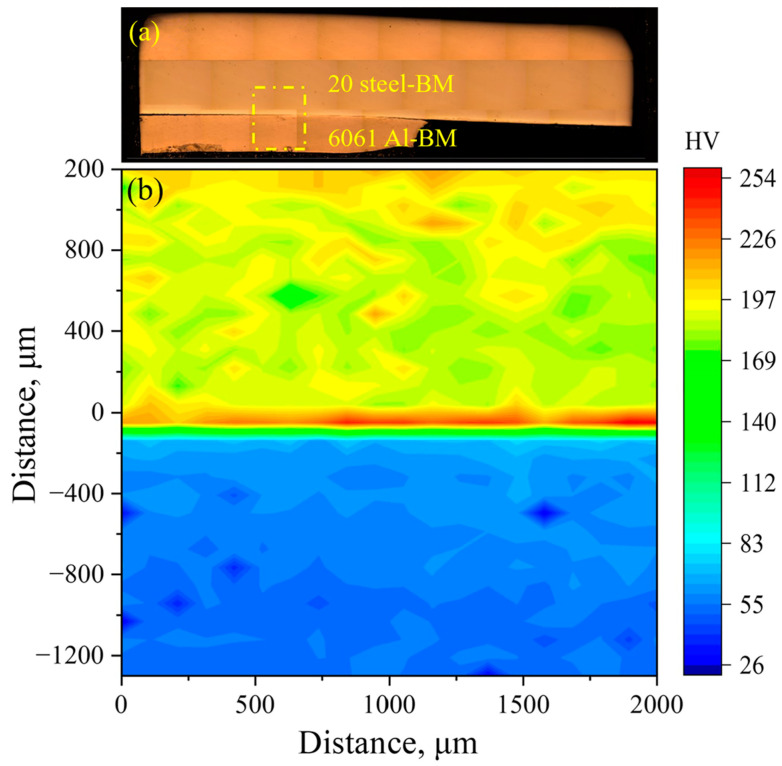
The microhardness at the region adjacent to the interface: (**a**) metallographical image of the whole joint; (**b**) the microhardness distribution of the marked zone in (**a**).

The microhardness test for the joint was performed on a digital microhardness tester with a dwell time of 5 s. The loads applied to the steel and aluminum alloy were 200 gf and 50 gf, respectively. The microhardness distribution in the region adjacent to the interface is presented in Figure 4. Figure 4a shows the practical position of the microhardness test. Obviously, the microhardness at the interface was greater than that on both sides, around 226 HV. In addition, the microhardness of 6061-BM near the interface increased slightly, and the closer it was to the interface, the higher the microhardness. Meanwhile, there was no obvious fluctuation in the microhardness of the 20# steel–BM. The work hardening resulting from intense collision during the welding process increased the microhardness at the interface and in the 6061-BM. However, this effect was negligible for the 20# steel–BM with relatively high microhardness. Considering the minimal change in microhardness between the regions near the interface and the BMs, only a narrow zone was affected during MPW.

**Figure 5:**
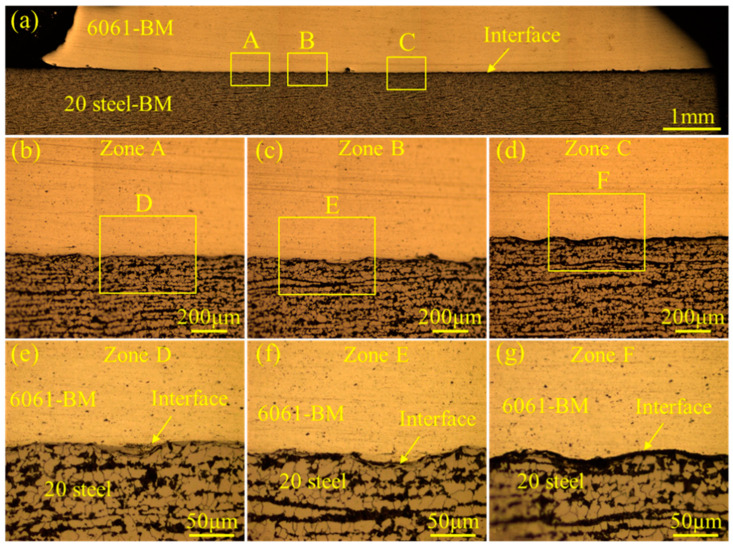
The metallographic microstructure at the bonding region of the 6061 aluminum alloy–20# steel welded joint: (**a**) the whole interface of the joint; (**b**–**g**) the microstructure of the interface.

The metallographic microstructure in the bonding region of the 6061 aluminum alloy–20# steel welded joint is displayed in Figure 5. No obvious welding defects, such as cracks or oxides, could be detected in the bonding zone (see Figure 5a). When traditional welding techniques (such as resistance spot welding (RSW) [15], melt inert-gas welding (MIG) [19] and laser welding [21], etc.) were employed to join the steel and aluminum alloy, brittle intermetallic compounds (IMCs) were inevitably formed in the interfacial zone. The formation of brittle IMCs could deteriorate the properties of the welded joints. However, as an innovative solid-phase welding method, MPW exhibited unique advantages in joining the dissimilar metals. Owing to the relatively low heat input, the growth of the IMC layer could be effectively restricted. As shown in Figure 5b–d, no IMC layer was detected at the interface. A different wave morphology was presented in Figure 5b–d. The relatively straight interface that formed at the beginning of the MPW process can be seen in Figure 5b,e. As the collision angle and velocity changed dynamically during the MPW process, the wavy interface exhibited a more significant amplitude (see Figure 5e–g).

## 4. Discussion

**Figure 6:**
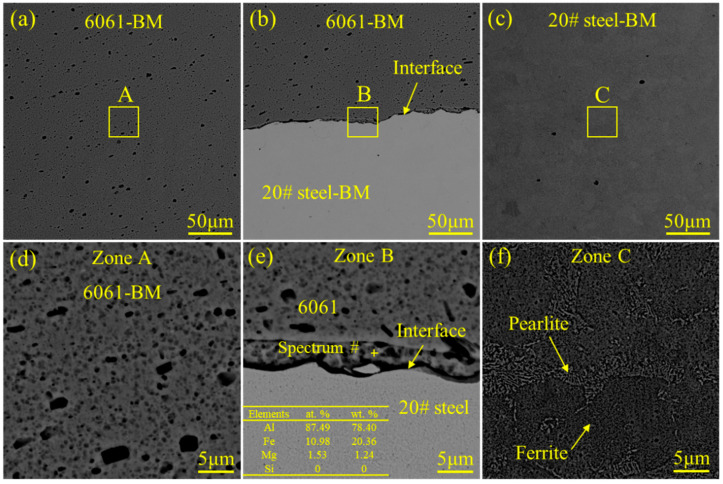
Microstructure of the characteristic zones for the 6061 aluminum alloy–20# steel welded joint observed by the SEM in back-scattered electron (BSE) mode: (**a**,**d**) the microstructure of the 6061–BM; (**b**,**e**) the microstructure of the interface between 6061 aluminum alloy and 20# steel; (**c**,**f**) the microstructure of the 20# steel–BM.

The SEM images of each characteristic zone for the 6061 Al alloy–20# steel welded joint are presented in Figure 6. The microstructure of 6061 aluminum alloy was α-Al, and dispersed precipitated phase particles were present in the 6061–BM (see Figure 6a,d). Wang et al. [41] also found similar precipitated particles in the 6061 Al alloy welded sheets, and deemed that the precipitated particles were Mg–Si intermetallic compounds. The microstructure of 20# steel was composed of ferrite and pearlite. The interface mainly consisted of elements Al and Fe, according to the energy dispersive spectroscopy (EDS) analysis.

**Figure 7:**
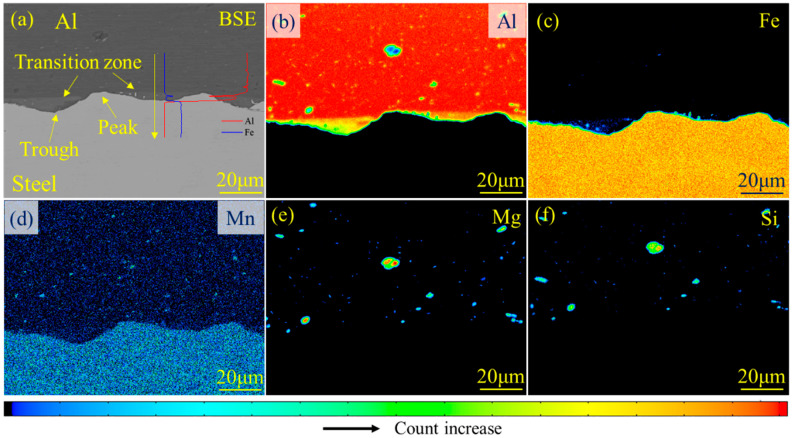
The element distribution in the interfacial zone of the MPW steel/Al tube: (**a**) the microstructure and EPMA line scanning results (the inside red and blue curves) of the interface zone of the steel/Al tube; (**b**–**f**) the element distribution of elements Al, Fe, C, Si, and Mg. A transition layer could be detected in the interface zone. Since the transition layer did not possess high hardness and brittleness like the Fe–Al binary IMC layer, the interface bonding quality of the MPW steel/Al tube was relatively good.

The element distribution in the region around the interface is presented in Figure 7. The dark region is Al–BM, and the bright region is steel–BM. The mixture distributed between the waves was considered as the transition zone. It is worth noting that no obvious transition zone was detected at the peak of the wave, and the thickness of the transition layer was almost equal to the wave height. Cui et al. [42] also detected the presence of a transition layer in the trough of the wave. The distribution of the transition zone was related to the element diffusion at the interface. It was deemed that the concave interface was more beneficial to the element diffusion, accounting for the formation of a thicker transition layer in the trough of the wave. Meanwhile, the simultaneous segregation of elements Fe and Al in the transition zone could be detected by consulting the line-scanning energy spectrum (see Figure 7a). The thickness of the transition layer was about 6 μm, based on the result of line-scanning. It was deemed that the transition zone might be composed of Fe particles detached from steel–BM during the MPW process and the Fe–Al solid solution according to the previous study of Wang et al. [37]. Since the transition layer did not possess high hardness and brittleness like the Fe–Al binary IMC layer, the interface bonding quality and long-term stability of the MPW steel/Al joint was relatively satisfactory. The MPW technique has been adopted by more and more automobile manufacturers, relying on its unique advantages in welding dissimilar materials, driving the development of lightweight automobiles [28]. Besides this, the corrosion resistance of the MPW steel/Al joint is significant in influencing the service behavior of the joint, yet this topic was not discussed here. Comparing the element distributions shown in Figure 7e,f, a similar distribution tendency between elements Si and Mg could be detected. The simultaneous segregation of elements Si and Mg was caused by the precipitation of the Mg_2_Si phase in the Al matrix. Yong et al. [43] deemed that the main precipitated phase of 6061 aluminum alloy was the Mg_2_Si phase.

**Figure 8:**
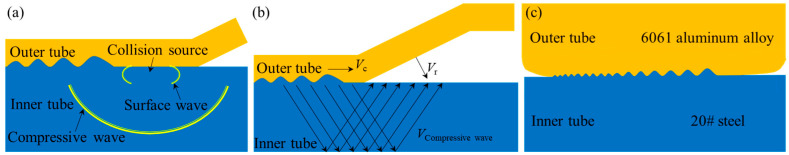
Schematic of interfacial wave formation at the magnetic pulse welded steel/Al tube: (**a**) elastic wave produced by collision; (**b**) compressive wave propagation during MPW; (**c**) interfacial morphology after welding. The wavy morphology of the interface increased the bonding area between the BMs (the corresponding experimental results are shown in Figure 7a–d), which was conducive to improving the mechanical properties of the joint.

Figure 8 presents the schematic of interfacial wave formation at the magnetic pulse welded steel/Al tube. *V_r_* refers to the impact velocity of the outer tube, and *V_c_* is the migration velocity of the collision point. According to the theory of stress wave, not only a compressive wave but also a surface wave was produced when the collision of two tubes occurred (see Figure 8a). The propagation velocity of the compressive wave was faster than that of the surface wave. In addition, their main propagation directions were also different. The compressive wave propagated inside the tube along the collision direction, while the surface wave propagated along the surface. Generated at the collision point, the compressive waves were reflected at the inner surface of the tube. Subsequently, the interface waves were generated via the interaction of refraction waves and the new compressive waves, as shown in Figure 8b. Ben-Artzy et al. [38] suggested that this phenomenon always occurred at and near the collision point. The presence of a surface wave could also lead to the appearance of a wavy interface, although these minor waves were flattened by subsequent collision. A wavy surface morphology away from the bonding area was detected in Figure 3e. The wavy interface formed without collision could prove the effect of the surface wave on the interface morphology.

The bonding of the steel/Al tubes occurred in the intermediate part of the interface, as shown in Figure 8c. In the initial stage of the MPW process, effective bonding could not be achieved without a suitable collision angle, although the velocity of collision between the steel and Al tubes was large enough. Dang et al. [44] also found that there was an unbonded zone with a length of about 1 mm in the initial collision zone. The velocity of the outer tube gradually decreased during the welding process. The metallurgical bonding between the steel tube and Al tube could not continue when the collision energy was less than its critical value. Mousavi et al. [45] kept the collision angle unchanged, and noticed that the effective bonding of the interface depended on whether the collision energy was high enough. In addition, the amplitude of the wavy interface increased gradually with the decrease in collision velocity and the increase in collision angle.

The bonding of the BMs was affected by the interfacial morphology, to some extent. As for the steel/aluminum MPW joints, the mechanical properties depended largely on the formation of the wavy interface. On one hand, the wave morphology of the interface increased the bonding area between the BMs. On the other hand, this waveform could achieve mechanical interlocking, effectively enhancing the bonding force of the joint. Thus, the formation of the wavy interface was considered as the crucial factor explaining the relatively qualified mechanical property of the MPW joint.

This study can not only offer a reference for the industrial application of the MPW steel/Al joint, but also clarifies the correlation mechanism between the microstructure and mechanical properties of the MPW steel/Al joint, providing guidance for practical application and also a strategy to improve the interfacial bonding quality of the joint.

## 5. Conclusions

The microstructure and interfacial morphology of the magnetic pulse welded steel/Al tubes were investigated. The elemental distribution in the interfacial zone was analyzed. Furthermore, the formation mechanism of the wavy interface was systematically discussed. The main conclusions are listed as follows:

(1) An interface with effective metallurgical bonding was successfully obtained at the intermediate part of the joint. No obvious IMC layer could be traced owing to the low heat input of the magnetic pulse welding (MPW), while the transition zone was distributed in the trough of the wave. That is to say, only element diffusion occurred during the MPW process. The thickness of the transition layer was approximately 6 μm;
(2) The interface of the as-welded joint could be divided into three zones—the bonded zone in the center, and unbonded zones on both sides. The formation of the wavy interface depended on the collision velocity and collision angle. The amplitude of the wavy interface increased gradually with the decrease in the collision velocity and the increase in the collision angle. Appropriately reducing the collision velocity and increasing the collision angle could improve the bonding quality of the interface;
(3) The 6061 aluminum alloy and 20# steel tubes were successfully joined by the MPW process. The qualified mechanical properties of the joint could be attributed to the formation of a wavy interface. The microhardness at the interface was higher than that on both sides, owing to work hardening, and was approximately 226 HV. In addition, precisely controlling the coil size and achieving a better alignment between the outer and the inner tubes could improve the properties of the welded joint;
(4) To further understand the relationship of the mechanical properties of the MPW joint with the specific interface microstructure, systematic analyses of the mechanical properties are preferred. Besides this, not only the joining between the tubes, but also the joining of specimens with other shapes, e.g., plates, can also be taken into account as attributable to the unique advantages of the MPW technique.

## Figures and Tables

**Table 1:** The chemical composition of 20# steel and 6061 aluminum alloy (wt. %).

Elements	Mg	Si	Cu	Cr	C	Ni	Mn	Fe	Al
20# steel	-	0.3	0.25	0.2	0.2	0.3	0.5	Bal.	-
6061	0.8–1.2	0.4–0.8	0.2	0.3	-	0.7	0.15	-	Bal.

## Data Availability

The original contributions presented in this study are included in the article. Further inquiries can be directed to the corresponding authors.
